# Hip arthrodesis in children: A review of 28 patients

**DOI:** 10.4103/0019-5413.55977

**Published:** 2009

**Authors:** Ashok K Banskota, Shikshya P Shrestha, Bibek Banskota, Binod Bijukacche, Tarun Rajbhandari

**Affiliations:** Hospital and Rehabilitation Centre for Disabled Children (HRDC), Kathmandu University Teaching Hospital, Jangal, Kavre, Nepal

**Keywords:** Hip arthrodesis, arthrodesis in children, cobra plate, post infective arthritis

## Abstract

**Background:**

The best method of treating intractable hip pain in an unsalvageable hip joint in a child is still a subject open to debate. We believe that hip arthrodesis in such patients provides a painless and stable hip for most activities of daily living in our challenging rural terrain. Therefore, we conducted this study to assess the functional ability of children with painful hip arthrosis treated by arthrodesis of the hip.

**Materials and Methods:**

A retrospective evaluation of 28 children (out of 35) who had an arthrodesis of the hip performed between 1994 and 2008 was carried out. The average age was 14 years, with 12 males and 16 females. There was involvement of the right hip in 13 and left in 15 cases. The average duration of follow-up was 4.87 years. The preferred position of the hip for arthrodesis was 20–30° of flexion, neutral abduction-adduction, and neutral rotation, irrespective of the method of fixation.

**Results:**

The average duration of clinical and radiological arthrodesis was found to be 4 months (2–6 months). At the last follow-up, all patients were painfree and had good ambulatory capacity. The average Modified Harris Hip Score increased from 53 to 84 and the average post-surgical limb length discrepancy was 1.3 cm, which was well tolerated in all cases. Patients, however, had difficulty in squatting and had to modify their posture for foot care, putting on shoes, etc. Also, some patients complained of ipsilateral knee, contralateral hip, or low back pain with prolonged activity, but this was not severe enough to restrict activity except in one case that was known to have juvenile rheumatoid arthritis and needed ambulatory aid.

**Conclusion:**

In an environment where pathology generally presents very late and often in a dramatic manner, where the patient's socioeconomic status, understanding, compliance, and the logistics of follow-up are consistently a challenge in management, hip arthrodesis has been an important procedure for our patient group, with good short-term results and promising midterm, and, hopefully, long-term prospects. In our series of patients, we have been successful in restoring painfree mobility.

## INTRODUCTION

The management of destructive hip pathology as a sequalae to sepsis, tuberculosis, inflammatory arthritis, chronic neglected trauma in the very young patient, continues to remain problematic. Joint replacement arthroplasty in the pediatric age group is not a commonly undertaken procedure because of higher failure rates and the potential need for multiple revisions. The goal of any surgery addressing painful hip pathology is to provide a pain free mobile and stable hip joint. While this issue is adequately addressed by a total hip arthroplasty (THA) in patients older than 50 years, but in very young patients with destructive hip pathologies, multiple revisions following THA (33-45%) have been reported.^1^,^2^,^3^ Hence THA as a primary procedure in this population needs reconsideration.^3^,^4^ Hip arthodesis has functioned remarkably well in this patient population as a salvage procedure in postarthritic and post injective hip pathology in young children.^5^,^6^ Hence, we conducted this study to assess the functional ability of children with painful hip arthrosis treated by arthrodesis of the hip.

## MATERIALS AND METHODS

**Figure 1:**
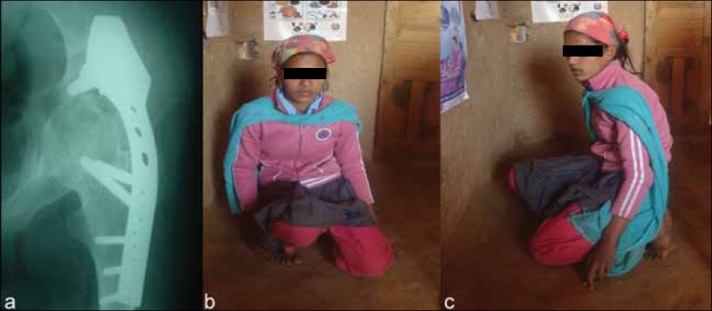
(a) Anteroposterior X-ray of left hip at 9.5 years follow up showing cobra plate *in situ* and successful fusion. (b,c) Clinical photograph at 9.5 years of same patient showing good function

**Figure 2:**
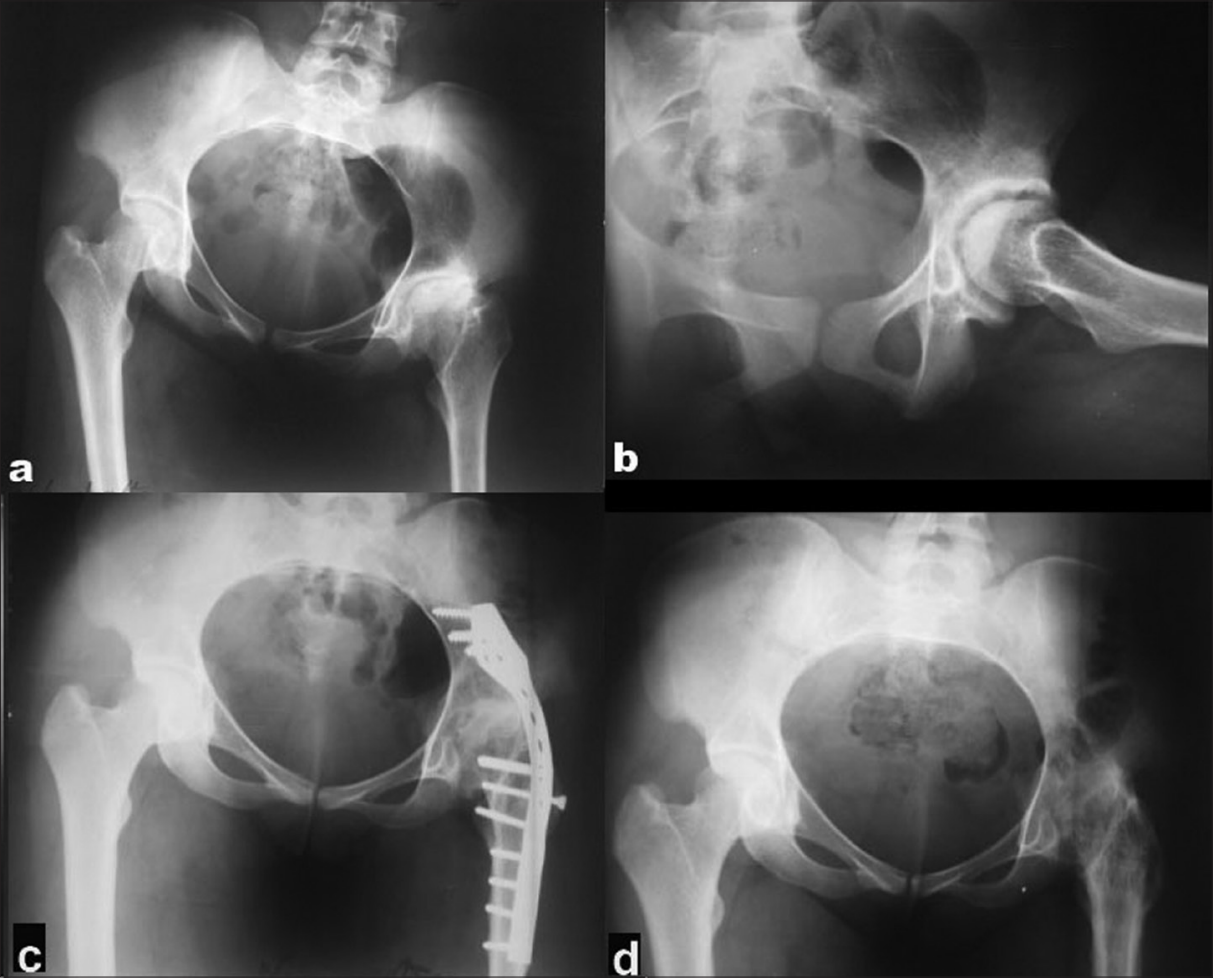
(a) Pre-operative X-ray of pelvis including both hips (anteroposterior view) and lateral view of left hip (b) showing post-infective hip with reduced joint space, coxa breva, and subchondral sclerosis on both femoral and acetabular sides. (c) X-ray of pelvis including both hips (anteroposterior view) showing Cobra plate fixation of the left hip with obliteration of the joint space. (d) Removal of implant showing trabecular bone growth across the joint at 10.5 years follow-up

A retrospective study of 28 patients was carried out from data obtained from patient records. Out of 35 children who had an arthrodesis of the hip performed between 1994 and 2008, 28 were available till the latest follow-up (2.5-10.5 years; average 4.87 years). The average age was 14 years, with 12 males and 16 females. There was involvement of the right hip in 13 and left in 15 cases. The average duration of follow-up was 4.87 years (2.5–10.5 years). The average age at which arthrodesis was performed was 14.36 years (range, 11–23 years). Twenty-three patients were between 10 and 15 years of age, four between 16 and 20, and one patient was 23 years old. There were 12 males and 16 females with 13 right- and 15 left-sided involvement. The agewise break-up of patients with their diagnosis, mode of treatment, radiological features, and follow-up is presented in the patient data [Table 1]. The average duration of symptoms was 3.17 years (4 months to 13 years). Tissue biopsy was sent for all cases at the time of surgery. The diagnosis was post-infective hip in 21 cases (three tubercular) [Figures 1 and 2], post-traumatic in three cases (two posterior hip dislocations and one chondrolysis leading to arthrosis following a fracture of the neck of femur), three chronic synovitis, and one juvenile rheumatoid arthritis. All post-infective hips were sequelae, of which three were biopsy-proven tuberculosis. In the remaining, we assumed a diagnosis of a post-infective sequel based on a history of fever, hip pain, limp, and/or a discharge in different combinations. Patients were operated at an average of 3.17 years (4 months to 13 years) of initial pathology [Table 1].

**Table 1:** Clinical details of patients

Age at arthrod-esis	Sex	Side	Presentation	Symptom duration (years)	Etiology	Approach	Method of fixation	Follow-up (years)	Pre-op MHHS^1^	Post-op MHHS*	Pre-op LLD †(cm)	Post-op LLD (cm)	X-ray findings
15	F	Left	Limp	9	Post-infective	Posterior	Screws (3)	4.5	39	88	2	1	AVN
15	M	Right	Pain, deformity	13	Tuberculosis	Anterolateral	Hip spica	6.5	46	80	1.5	1	Dislocated hip, sclerotic head
16	F	Left	Pain, limp	5	Post-infective	Posterior	Cobra plate	3	58	80	4	2	Secondary arthrosis
13	M	Left	Pain, limp, deformity	0.67	Non-union NOF‡	Anterolateral	DCP§	4.5	69	87	2.5	1	Secondary arthrosis
16	F	Left	Pain, limp, stiffness	0.33	Tuberculosis	Posterior	Cobra plate	5	52	88	4	2	Dislocated left hip
14	F	Left	Pain, limp	9	Post-infective	Anterolateral	Cobra plate	4.5	69	91	0.5	0.5	Secondary arthrosis, coxa magna
14	F	Right	Pain, limp, stiffness	4	Post-infective	Posterior	Cobra plate	3	50	88	1.5	1	Secondary arthrosis
23	M	Right	Pain, deformity	1	Post-infective	Anterolateral	DCP	10.5	39	86	2	1	Secondary Arthrosis, coxa magna
13	F	Right	Pain, limp, stiffness	5	Chronic synovitis	Anterolateral	Screws (3)	7.5	46	81	1.5	1.5	Secondary arthrosis
12	M	Left	Pain, swelling, limp	2.5	Post-infective	Anterolateral	Threaded pins (3)	5.5	50	82	4	2	Dislocated hip, destroyed head, dysplastic acetabulum
15	F	Right	Pain, swelling, limp	2	Post-infective	Anterolateral	DCP	9.5	41	88	1.5	2	Secondary arthrosis
11	F	Left	Pain, deformity	2.5	Post-infective	Posterior	Hip spica	5.5	40	84	2	2	Secondary arthrosis
14	F	Left	Pain, limp	2	Chronic synovitis	Posterior	Cobra plate	3	74	83	1	1.5	Secondary arthrosis
13	F	Right	Pain, limp, deformity	0.83	Post-infective	Posterior	Cobra plate	2.5	74	88	0	0	Secondary arthrosis
11	F	Right	Pain	1	Post-infective	Posterior	Cobra plate	2.5	54	76	2	2	Secondary arthrosis, coxa magna
13	F	Left	Pain, deformity	0.58	Post-traumatic	Posterior	Cobra plate	2.5	45	86	2	2	AVN femoral head
15	F	Right	Pain	3	Post-traumatic	Posterior	Cobra plate	3.5	51	86	1	1	AVN femoral head
15	M	Left	Pain	5	Post-infective	Anterolateral	Cobra plate	6.5	61	86	0	0	Fibrous ankylosis
16	M	Right	Pain, swelling	1	Post-infective	Posterior	Cobra plate	3.8	58	85	2	1.5	Secondary arthrosis
14	M	Left	Pain, limp	3.5	Chronic synovitis	Posterior	Cobra plate	6.5	50	80	1.5	1	Secondary arthrosis
16	F	Right	Pain, swelling	2	Post-infective	Posterior	Cobra plate	5.5	46	77	2	1.5	Secondary arthrosis
15	M	Left	Pain, limp	1.25	Post-infective	Posterior	Hip spica	2.5	68	88	2	1.5	Secondary arthrosis
14	M	Right	Pain	0.83	Tuberculosis	Anterolateral	Screws (3)	2.5	75	80	2	1	Secondary arthrosis
12	M	Left	Pain, limp	1	Post-infective	Posterior	DCP	3.5	64	80	3	1.5	Secondary arthrosis
13	F	Left	Limp, deformity	6	Post-infective	Posterior	Cobra plate	3.5	50	89	3	2	Secondary arthrosis
15	M	Left	Pain, discharge	3	Post-infective	Posterior	Hip spica	6.5	44	72	3	2	Secondary arthrosis
15	M	Right	Pain multiple joint	3.5	JRA**	Posterior	Cobra plate	7.3	38	62	2	2	Arthritis
14	F	Right	Pain, swelling	0.33	Post-infective	Anterolateral	Hip spica	4.8	53	83	2	1.5	Secondary arthrosis

* Modified Harris hip score,

† Limb length discrepancy,

‡ Neck of femur,

§ Dynamic compression plate,

** Juvenile rheumatoid arthritis

The average follow-up was 4.87 years (2.5–10.5 years). The main indication of surgery was painful hip pathology restricting ambulation and activities of daily living (27 patients) alone or in combination with severe deformity that did not permit normal gait. Posterior approach was used in 18 cases and anterolateral in 10 cases, of which 15 were fixed with a cobra head plate [Figure 1], four with a dynamic compression plate, three with cannulated screws, one with threaded pins, and five with a hip spica. We preferred the posterior approach in patients with significant bone loss and deformity necessitating extensive dissection and when the possibility of future arthroplasty was not contemplated. The approach used was also a matter of surgeon preference. An anterior swelling necessitating synovectomy led to preference of the anterolateral approach in five cases, (one chronic synovitis, one tuberculosis and three post-infective some cases). The patients with fixation devices were kept non-weight bearing for 6 weeks followed by progressive to full-weight bearing at 3 months. Those treated in a hip spica alone were immobilized non-weight bearing for 3 months. Thereafter, patients were seen at 3-monthly intervals for the first year and then at 6-monthly intervals. The arthrodesis was labeled successful if, clinically, the patient was painfree and was able to walk without any pain or limp and, radiologically, if there was obliteration of the joint space and trabecular bone growth across the joint [Figure 2].

In our series, the preferred position for arthrodesis was 20-30° of hip flexion, neutral abduction/adduction, and neutral rotation. We preferred this position because we succeeded in achieving a leg length discrepancy (LLD) of less than 2 cm in all our patients and subsequent follow-ups did not reveal any problems as this LLD was very well tolerated by all patients.

All patients completed clinical and radiological evaluation along with completion of a standard questionnaire related to pain and function (Modified Harris Hip Score, MHSS) and some additional questions relating to quality of life post surgery. The pre-operative MHSS was compared with the post-operative MHSS at the latest follow-up.

## RESULTS

Majority of the hips selected for arthrodesis had an infective etiology (21 cases, including three tubercular) followed by trauma (three) and chronic synovitis (three) and juvenile rheumatoid arthritis (one). The post-traumatic cases included two untreated posterior hip dislocations and one arthrosis secondary to chondrolysis following an untreated fracture of the neck of femur. The major presenting symptom was pain (27 patients) followed by deformity of the hip. Thirteen patients had a fixed flexion deformity ranging from 40° to 76° degrees, six patients had fixed abduction ranging from 5° to 30°, five had fixed adduction ranging from 10° to 20°, and five patients each had fixed internal and external rotations ranging from 5° to 20°. Fifteen hips were fused using a cobra plate device and five hips were allowed to fuse in a functional position in a hip spica following decompression, debridement, and curettage of the articular surfaces, four were fixed with a dynamic compression plate, three with cannulated screws, and one with threaded pins. The main determinants of the method of arthrodesis were bone stock and size of the child. Generally, the aim was to achieve secure fixation to maintain the position of fusion, which was adequately addressed using a cobra head plate in most cases. However, in cases where bone quality was poor or fixation with cobra plate was not feasible, e.g. in very small-sized hips, other implants were used or it was decided to achieve arthrodesis in a hip spica. Pre-operative radiographs showed a secondary arthrosis in 20 patients.

The average duration of clinical and radiological arthrodesis was found to be 4 months (2–6 months). A fibrous pseudarthrosis developed in one patient, but this was pain free. At the last follow-up, all patients were painfree and had unlimited ambulatory capacity. Functional outcome was not significantly different for various geographical regions from where the patients came, e.g. patients living in the plains fared as well as those living in hilly areas.

At the latest follow-up [mean, 4.87 years; range, 2.5–10.5 years], 21 patients did not have any limitations in any activities of daily living while three patients had difficulty carrying heavy loads and four said that they could not walk as fast as their colleagues. All patients had to modify their posture for squatting for toileting purposes, wearing lower body garments, foot care, and for putting on socks and shoes.

Also, three patients had contralateral hip pain, one had ipsilateral knee pain, and two had contralateral knee pain with long distance walking, while three patients had low back pain. The average duration of development of these symptoms was about 4 years following the hip arthrodesis. Only one patient needed a gait aid (crutch) to cope with his ipsilateral knee and contalateral hip pain. This patient had a history of juvenile rheumatoid arthritis and his symptoms probably reflect the natural history of his condition rather than be attributable to the arthrodesis. Three patients developed an adduction drift of 5° to 10° over 3 years. This phenomenon was seen only in patients who had hip arthrodesis at a younger age (age 11–13 years) than the average.

The average pre-operative MHHS was 53 and the average post-operative score was 83. The average limb shortening was 1.3 cm, which was well tolerated by all patients.

## DISCUSSION

A painfree, mobile, and stable hip joint is the goal of any surgery addressing painful hip pathology. While this adage is addressed adequately by total hip arthroplasty (THA) in patients over 50 years of age, the long-term durability of implants and need for multiple revisions with their potential problems limit their applicability in very young patients with painful destructive hip pathology.^1^ This, coupled with reports of very high revision rates (33–45%) of THA in this population highlights the need to reconsider THA as the primary procedure in this patient group.^2^–^4^ Hip arthrodesis, as a bone conserving surgery, stands between the options of non-surgical modalities (cane, analgesics) or a THA, with advantages of pain relief, possibility of an active lifestyle, and the option of conversion to THA later on.

Early reports on hip arthrodesis with internal fixation reported fusion rates of 74–78%, with the surgical technique being the most important factor influencing outcome.^5^,^6^ In our series, fusion was achieved in 27 hips and a fibrous pseudoarthrosis developed in one patient. At the last follow-up, 21 patients reported that they did not have any limitation in any activity of daily living while three patients had difficulty carrying heavy loads and four said that they could not walk as fast as their colleagues. All our patients required modification in traditional toileting practices for which squatting is necessary and also reported difficulty in putting on shoes and socks, as reported in some other series.^7^,^8^

Hip arthrodesis should be considered in high-demand young patients without pre-existing lumbar or ipsilateral knee or contralateral hip arthritis, with monoarticular disease, in whom a THA is not yet justified. Polyarticular disease or symptomatic developmental hip dysplasia should not be considered for arthrodesis because of the rapid development of contralateral symptoms and arthritic changes.^9^

The most important factor determining the success of arthrodesis of the hip is the position of fusion in three planes.^9^ Several authors have accepted flexion between 15° and 40°, 0° to 10° degrees of adduction^10^ or abduction^5^ and 0° to 10° of external rotation.^11^,^12^ The optimal position for hip fusion seems to be 20-30° flexion, 5° of adduction, and 5°–10° external rotation with minimal limb length inequality, correction through leg adduction (shortening) or abduction (lengthening) being limited to a 2-cm difference.^9^

The assessment of hip arthrodesis is dependent on the functional outcome and the effect on adjacent joints (contralateral hip, ipsilateral knee, and lower back). In their study of 200 patients with unilateral hip fusion at an average follow-up of 22 years, Hauge *et al*. noted radiological osteoarthritic changes in 65% of the patients, with 20% complaining of knee pain or instability.^13^ Other authors have noted a painful ipsilateral knee and low back in 57–61% of the patients and correlated this finding with malpositioning of the fused hip (excessive abduction).^14^,^15^ Gudmundsson *et al*. reported on 125 patients with fused hips at 10 years and found that patients with mobile hips showing propensity for arthritic deterioration (e.g. developmental hip dysplasia) had poorer functional outcome.^16^ In our series, one patient needed crutches to cope with her ipsilateral knee and contralateral hip pain. Two patients in our series had contralateral knee pain at the last follow-up (6.5 years and 9.5 years follow-up respectively). The options available to tackle this problem are to either convert a fused hip to a THA or carry out arthroplasties of other affected joints if the position and function of the fused hip are satisfactory. Although the latter option yielded unpredictable results in several reported series, it could still be the dominant option in a patient with a hip fused in a satisfactory position but with questionable abductor function.^17^–^19^

Results of conversion of a fused hip to a THA, to address ipsilateral knee, contralateral hip, or lower back pain, are inferior to that of a primary THA and have a higher complication rate. Hips that fused spontaneously seem to perform better following THA than surgically fused hips as do conversions in patients older than 50 years compared with younger patients. The higher rates of infection may reflect the history of infection. The pre-arthroplasty status of the abductor muscles is a very important factor, so much so that some authors feel that a conversion should not be performed if adequate abductor function is not present. This is best evaluated pre-operatively by palpation of the contracting abductor muscles.^20^–^24^

In conclusion, hip arthrodesis is a very effective way to deal with painful hip pathology limiting function in young patients with an option of conversion to a THA at a later stage. The most important factor warranting such conversion seems to be the development of contralateral hip, ipsilateral knee, and lower back pain in the long term.

In our setting, where a vast majority of the population lives rurally in socioeconomically constrained circumstances coupled with increased predisposition and decreased access to healthcare following infection, trauma etc., this procedure has been very effective in making our patient group ambulatory and giving them a chance to develop socioeconomically alongside their peers in a socially respectable way. In our own patient population (rural background), hip arthrodesis has functioned remarkably well as a salvage procedure in the post-infective or post-arthritic hip in young children. Our results are encouraging enough to recommend the continued application of this durable and effective procedure both in the short- and long-term contexts. We believe that hip arthrodesis leads to excellent functional gains in children suffering from painful hip pathology otherwise limiting them in their capacity to ambulate and activities of daily living.
